# Psychological well-being, stressors, coping strategies and support of undergraduate healthcare students amid COVID-19

**DOI:** 10.4102/hsag.v28i0.2340

**Published:** 2023-12-08

**Authors:** Annali E. Fichardt, Corlia Janse Van Vuuren, Lynette van der Merwe

**Affiliations:** 1 Department of Nursing, Faculty of Health Sciences, University of the Free State, Bloemfontein, South Africa; 2 Department of Health and Rehabilitation Sciences, Faculty of Health Sciences, University of the Free State, Bloemfontein, South Africa; 3 Division Health Sciences Education, Faculty of Health Sciences, University of the Free State, Bloemfontein, South Africa

**Keywords:** psychological well-being, stressors, coping strategies and support, undergraduate healthcare students, COVID-19

## Abstract

**Background:**

Students function better academically when psychologically well. The COVID-19 pandemic proved a new challenge to the mental wellness of undergraduate healthcare students. Students were not only faced with academic changes but also had to complete clinical practice in healthcare facilities amid the COVID-19 pandemic.

**Aim:**

This study investigated the psychological well-being, stressors, coping strategies and support of undergraduate healthcare students amid COVID-19.

**Setting:**

A South African university’s faculty of health sciences.

**Methods:**

A survey design through a cross-sectional descriptive approach was used to collect data from the population of 1529 undergraduate healthcare students. One hundred and ninety-six (*n* = 196) students responded to the online survey.

**Results:**

Participants reported a variety of stressors influencing their psychological well-being. Participants chose mostly adaptive coping strategies to deal with stressors. They gave feedback on the support they received from the faculty and institution. Most of the participants indicated they prefer weekly online communication from the higher education institution.

**Conclusion:**

The COVID-19 pandemic influenced the psychological well-being of undergraduate healthcare students. The psychological well-being of these students is a collective responsibility between students and higher education institutions to enable academic success and positive patient outcomes.

**Contribution:**

This study found that undergraduate healthcare students had academic, psychological, financial and other stressors during the COVID-19 pandemic. Higher education institutions, especially those involved in training undergraduate healthcare professionals, in collaboration with students, need to provide students with targeted continued support and training to use healthy coping behaviours to manage various stressors.

## Introduction

The psychological well-being of students, specifically healthcare students, is well researched and remains an area of concern. Shortly before the outbreak of the COVID-19 pandemic, Auerbach et al. (2018:17) reported in a World Health Organization (WHO) World Mental Health Survey that one-third of first-year college students screened positive for mental health problems. This finding represents a critical global mental health and higher education issue as excessive and unconstructive mental distress is directly associated with lower academic performance (Bruffaerts et al. 2018:9), a poorer overall health state and even psychological distress, with symptoms of anxiety and depression. Continued psychological distress among students could lead to academic burnout or a state of emotional exhaustion, which results in students experiencing emotions of decreased personal achievement and disinterest in their studies, further negatively impacting academic learning (Lin & Huang 2014; Tomaschewski-Barlem et al. 2013). A lack of psychological well-being, as reported on in this study, includes signs of perceived anxiety and depression among students, leading to continued psychological distress and the subsequent effects as described in this paragraph.

Student mental health issues, such as anxiety and depression, have escalated from existing crisis levels before the COVID-19 pandemic (Visser & Law-Van Wyk 2021:239). In their study, Wieczorek et al. (2021:9) found that nearly 77% of students presented with psychopathological symptoms using the General Health Questionnaire (GHQ-28) during the COVID-19 pandemic. While Visser and Law van Wyk (2021:229) in their study among undergraduate students from various faculties, found that 45.6% and 35% of participants referred to experiences of anxiety during the pandemic. The adverse psychological effects of COVID-19 are well described. Mental health issues were evident in healthcare students as they not only had to adjust to the changes in their academic environments such as studying without the support of an academic environment but were more exposed to the pandemic because of the required clinical work they had to do in the clinical facilities (Wald 2020:744). During the COVID-19 pandemic, healthcare students were subjected to different academic (including practical and clinical training) arrangements, each requiring its own adjustment, based on the national alert levels (Department of Health 2020:4). During the hard lockdown that lasted from 27 March to the end of April 2020, all students were studying online from their own private residences, with no practical and/or clinical training and no access to the academic institution. After initially adapting to the hard lockdown, these students had to re-adapt to changed academic programmes, including changes in practical and/or clinical work, changes in living arrangements away from campuses and changes in their personal lives upon returning to campuses early after the hard lockdown and before any non-healthcare students returned to campus (Connolly & Abdalla 2022:1; Gordon et al. 2020:1202). Therefore, healthcare students experienced even greater stress, a feeling of losing control and continuous uncertainty (Wald 2020:1). Support provided during these initial phases, before the return of non-healthcare students to campus, was of vital importance in healthcare faculties.

Support structures for higher education students are widely described and can be divided into academic support (Hoyne & McNaught 2013:109), emotional support (Regehr, Glancy & Pitts 2013:1), or integrated academic and emotional support through mentoring (Gershenfeld 2014:365). For this study, support was seen as overarching to include strategies or aspects in any of the three domains, namely, academic, emotional or integrated support. Because of the nature of stressors experienced by healthcare students during clinical training, emotional support structures for these students are more frequently described in literature than pure academic support structures. This, however, does not imply that healthcare students do not need or could not benefit from academic support and mentoring. Amid COVID-19, the emotional support of healthcare students became even more important, and healthcare educators were responsible for fostering resilience and well-being among their students (Wald 2020:1).

Based on observation at the university where this study was conducted, supports such as formal and informal communication from the institution through online platforms, WhatsApp messages from lecturers and online wellness programmes were in place, trying to address all three domains of support, as mentioned earlier. Students in this study also reported using a range of coping strategies, such as planning and prioritising, active coping, self-distraction or making time for leisure activities, avoidance, problem-solving and emotional support (Pereira & Barbosa 2013:1; Sreeramareddy et al. 2007:1). A previous study reported that medical students who use adaptive coping strategies have higher resilience scores (Van der Merwe, Botha & Joubert 2020:1). However, at the health faculty site of this research, a surge in anxiety levels among healthcare students became evident through the increased number of emails received from students, the requests and questions raised during online contact sessions and the increasing demand of individual student consultations with academics and psychological support staff as reported to the programme directors and heads of schools. This study therefore aimed to investigate undergraduate healthcare students’ psychological well-being, stressors, support needs and coping strategies amid COVID-19. Insight into their identified well-being could provide guidelines for interventions to enhance academic learning.

## Research method and design

A survey design through a cross-sectional approach was used, allowing for swift data collection (Creswell & Creswell 2018:211). This descriptive observational design is used to collect data on outcomes and exposure of a study population at a specific time (Wang & Cheng 2020:585). The online survey, including closed and open-ended questions, provided quantitative and open-ended question responses on the psychological wellbeing, stressors, coping strategies and support needs of undergraduate healthcare students amid COVID-19.

The study was conducted among undergraduate healthcare students at a faculty of health sciences at a South African university after returning to campus after the national COVID-19 hard lockdown in May 2020 was recalled. By applying the census sampling method, all 1529 registered undergraduate healthcare students in the faculty were invited to participate via the Blackboard learning management system and WhatsApp in the second semester of 2020. An information letter and the link to the EvaSys online questionnaire were included in the invitation to participate in the research. EvaSys is an online automated survey platform allowing for the development and administration of electronic surveys (https://evasys.de/en/). Three reminders were sent over six weeks to encourage participation.

The questionnaire was designed by the researchers using literature to address the study objectives. An information technology consultant assisted with the online survey administration. The questionnaire was in English and consisted of five sections. The first section obtained demographic information regarding the participants. This included the student number (voluntary and requested to assist with student referral if support needs were identified), degree the student was registered for, the academic year of study, clinical training facility the student was allocated to and duration of clinical work since the student had returned to campus after COVID-19 hard lockdown. The second section included open-ended questions on emotional support. In this section, participants were asked to reflect on their emotional experiences related to online learning and the clinical environment during the COVID-19 pandemic; the support, including communication on the support they received from the institution, faculty and departments; and the additional support required. Section three enquired about coping strategies used during and before the pandemic and stressors related to the COVID-19 pandemic (open-ended questions). Section four (closed questions) obtained data about the mode and frequency students wanted to receive from the university, faculty and departments. The fifth and final section of the questionnaire included the Brief COPE, a 28-item validated instrument measuring 14 coping scales to self-report coping strategies (Carver 1997:96). The Cronbach’s alpha score for the Brief COPE in a two-factor second-order model ranged from 0.81 to 0.88 (Rahman et al. 2021:1). For each scale, two statements reflecting a coping strategy are rated on a Likert scale of 1–4. The total for each scale is scored from 2 to 8. A higher score indicates greater use of this coping scale. The researchers confirmed the content validity of the questionnaire by using three rounds of round-robin and an online pilot by the researchers. The data from the pilot study were not included in the main study.

Before commencing the data collection, ethics approval was obtained from the health sciences research ethics committee at the institution. Gatekeepers’ approval was also obtained from the dean of the faculty, heads of departments in the faculty and the vice-rector of research and academics at the university. An information letter was part of the online questionnaire and email invitation. The consent was inferred by voluntary completion of the online questionnaire, and participants were informed that they could withdraw from the study at any time. Participants were offered an opportunity for psychological or academic support assistance if they experienced emotional distress or anxiety by participating in the study.

## Data analysis

After the online completion of the EvaSys survey, descriptive statistics, including frequencies, average scores, means and standard deviations, were generated by the EvaSys system to describe the quantitative data.

The EvaSys system also provided verbatim responses to open-ended questions. These open-ended question responses were transferred to an Excel spreadsheet by a research assistant. The open-ended question responses were then read and re-read by the researchers after the quantitative data analysis was completed. Through this process, the researchers aimed, firstly, to gain an in-depth understanding of the psychological well-being, stressors, coping strategies and additional support needed by healthcare students and, secondly, to gain a more in-depth understanding of quantitative data. Open-ended question responses were, thus, triangulated with findings from the quantitative data using illustrative quotes, as included in this article.

### Ethical considerations

Approval for the study was obtained from the Health Sciences Research Ethics Committee, University of the Free State (UFS-HSD2020/1376/2909). Informed consent was obtained from all participants.

## Results

Of the 1529 registered undergraduate healthcare students included in the study, 196 students participated (with an overall response rate of 12.8%). Of the participating students, 51% were from nursing (NS); 27% were from disciplines of physiotherapy (PS), occupational therapy (OTS), optometry (OS), dietetics (DS), biokinetics (BS) and sport coaching (SCS); and 22% were from medicine (MS).

**FIGURE 1:**
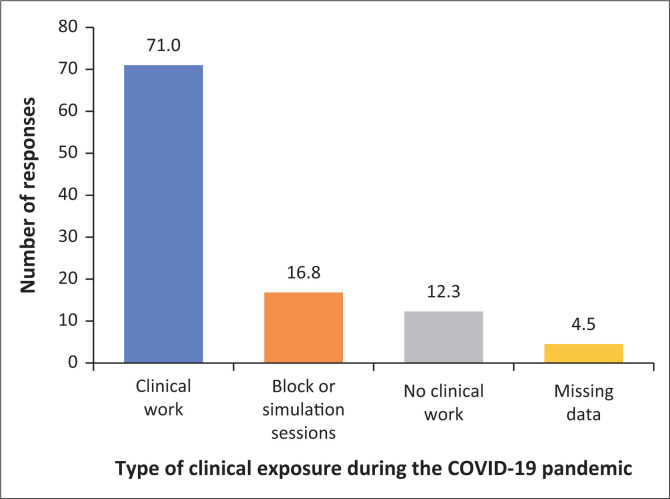
Clinical exposure of undergraduate healthcare students during the COVID-19 pandemic after the hard lockdown.

About a third of participants (*n* = 61, 31%) were in the fourth or higher year of study, with 50 (25.5%) in year three and 42 (21.4%) each in years one and two. This demographic profile of students held significance during the COVID-19 pandemic. Firstly, students returned to campus in a phased approach according to the alert levels, with final year students returning first to commence with clinical work within various healthcare settings. Secondly, clinical and/or practical training was adapted for some year groups, as fewer students engaged with clinical work because of access restrictions in many of the healthcare facilities; as well as practical training that had to take place in a smaller group setting. For more junior students, physical clinical engagement within the healthcare settings was replaced with paper-based case studies and/or simulation. Finally, the limited clinical exposure for pre-clinical students, to familiarise themselves with the clinical healthcare settings in preparation for their clinical study years, was cancelled. Clinical work refers to students’ clinical exposure in the work environment. Clinical work increases with the year of study. The findings in Figure 1 demonstrating the participants’ clinical exposure after the COVID-19 pandemic hard lockdown confirms the demographic profile of the participants, with most being in the clinical years of study.

One hundred and thirty-nine participants (71%) reported doing clinical rotations at training sites in a variety of clinical facilities ranging from primary healthcare settings, labour wards and psychiatric units to intensive care units at the time of completing the questionnaire. In contrast, a smaller number of students were in block or simulation rotations, and some students were not involved in any clinical work. Most participants (*n* = 79, 40.3%) reported completing 1–9 weeks of clinical rotations, while a slightly smaller percentage of participants (*n* = 77, 39.2%) reported completing more than 10 weeks of clinical rotations since returning to campus after the hard lockdown period. Participants who indicated that they were not exposed to clinical work during the COVID-19 pandemic were first-year undergraduate healthcare students who were not placed in clinical facilities yet and were still busy with pre-clinical training.

The next sections of this article, reporting on the cross-sectional descriptive study, offer insight into the undergraduate students’ psychological well-being, stressors and coping strategies in various healthcare programmes, including nursing, medicine, health and rehabilitation sciences, across 5 years of study.

### Psychological well-being and emotional experiences during the COVID-19 pandemic

Responses regarding emotional experiences linked to teaching and learning and clinical learning during the COVID-19 pandemic varied greatly. Even though the change to online learning created anxiety among some students, others saw the opportunities and their learning structure (see Table 1). Undergraduate healthcare students returned to campus before students from other non-healthcare faculties at the institution, to complete clinical rotations. Similarly, with regard to the online experience, some students managed to report on the positive experience, while other students gave negative feedback. Table 1 illustrates the contrasting views indicating positive or negative feedback by participants regarding their emotional experiences of online learning and clinical rotations amid the pandemic. Verbatim quotes from participants’ responses to open-ended questions in the questionnaire support these experiences.

**TABLE 1:** Emotional experiences feedback.

Emotional experiences	Positive feedback	Negative feedback
**Within the COVID-19 online teaching and learning environment**	‘I feel positive. It feels great to be able to continue with my studies, even though COVID-19 is still an important issue in our lives. It feels good knowing that I can spend my time on something meaningful.’ (MS 101)	‘High levels of stress and anxiety. The loss of the face-to-face component of learning was a big adjustment and not without many challenges. I think the stress and anxiety is partly due to not being able to feel part of a class group. As far as possible one tries to maintain that feeling of being one part of a whole but the online learning component has taken away that aspect or feeling. Now one has to dig deep and find internal sources of motivation. This is especially difficult for those who draw motivation and inspiration from others.’ (MS 159)
	‘I find it a nice alternative for us to keep on learn [*sic*] this way, and I find it comforting to know there are other ways that our programme can be adapted to meet our needs.’ (PS 24)	‘I find it very challenging to keep up to date with my work. I feel unmotivated and quite frankly depressed. I enjoy my studies, but my motivation to do my work on time is lacking and I have no idea how to change it. This is causing a huge amount of stress for me.’ (MS 192)
**Within the COVID-19 clinical environment**	‘Being back improved my emotional state immensely.’ (PS 4)	‘As much as we have PPE it causes a lot of anxiety knowing you’re in a hospital setting putting your life and others at risk because it is expected of hospitals to have a higher viral load.’ (OS 158)
	‘I feel we are being protected and kept safe as much as one could expect when working in a clinical environment. And on the other hand I feel like this is our job and what we would have had to deal with if we were qualified already, so to be given the experience of working during a pandemic now will only prepare us for our future lives.’ (PS 24)	‘I am afraid because in the trauma ward we treat patients that cough on us, shout in our faces because of pain, spit etc. before Covid tests are done. When we leave we usually do not get to see the results which causes stress. I am not afraid for myself, I go home to parents with co-morbidities.’ (MS 106)

PPE, Personal Protective Equipment.

### Stressors and coping strategies

Students had the option to indicate more than one stressor. Most students reported academic stress (*n* = 142, 72.4%), with 121 (61.7%) reporting psychological and 93 (47.4%) reporting financial stressors. Eighteen (9.5%) participants reported other stressors, including interpersonal (relationship problems), physical health and spiritual challenges as churches were closed.

Participants’ coping strategies were investigated using the Brief COPE questionnaire. Open-ended question responses reported about participants’ current coping strategies and how they differ from strategies used before the COVID-19 pandemic.

In this study, Brief COPE scores of equal to higher than a score of 3 (‘a medium amount’), to a score of 4 (‘a lot’) were seen for coping strategies including acceptance, planning and religion. Scores below 2 were equated with denial and substance abuse.

Open-ended question responses supported the self-reported coping strategies where ‘I set out an hour a day to exercise which helps me relax’ was aligned with the scale ‘Active Coping’, while ‘I blamed myself for all my problems’ was equated with the scale ‘Self-blame’.

The open-ended question responses revealed that participants employed various coping strategies to function during unfamiliar circumstances, including adaptive coping strategies. Examples included communicating with loved ones, friends, lecturers and fellow students (seeking emotional support), journaling, practising religion, meditating (religion), sleeping, physical exercises, working diligently and consistently, doing everyday chores, keeping to a strict timetable (active coping) and watching television (self-distraction). Examples of participants’ responses to open-ended questions regarding their coping strategies are as follows:

‘Introspection, personal reflection and debriefing with friend or family, or in the form of writing or meditation – Physical exercise, Attention to my surroundings controlling what can be controlled, like doing the dishes, and letting what can’t be controlled run its course – like this pandemic.’ (MS, Participant 195)

‘Exercise and sleep. Obviously there is a lot of work to do but I particularly feel relaxed after a run and I feel a lot less stressed during the day when I have gotton enough of sleep the previous night so I have been focusing on that mainly. I also speak to my mom for long periods over the phone or FaceTime to catch up and touch base with home.’ (PS, Participant 17)

**FIGURE 2:**
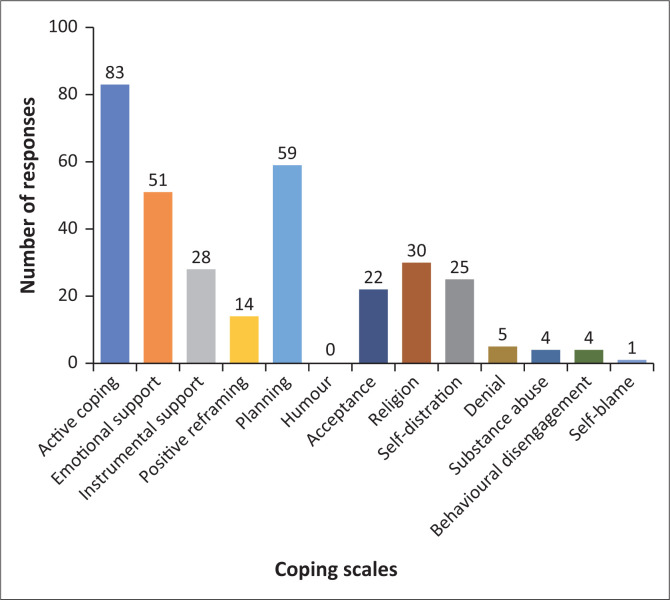
Participants’ responses to coping strategies.

Figure 2 triangulates participants’ responses to open-ended questions about their coping strategies with the Brief COPE scales. From the open-ended question responses, most participants described using adaptive coping strategies, triangulating with the Brief COPE scales, such as; active coping, planning and seeking emotional support, followed by religion and seeking instrumental support. The most described maladaptive coping strategies were; self-distraction, denial, behavioural disengagement or substance abuse (specifically cigarette smoking) described by very small numbers of participants. No participants described using humour as a coping strategy, and eight participants did not respond to the open-ended questions.

The most commonly mentioned maladaptive coping strategies were blaming themselves for all their problems, crying to release the pressure, eating unhealthily, drinking up to 10 cups of coffee daily and drinking medication. One participant reported alcohol binge drinking once a week.

About a third of participants (*n* = 63, 32%) indicated that their coping mechanisms during the COVID-19 pandemic did not differ much from before the pandemic. Support is provided from the open-ended response:

‘The truth is there is no difference between now and in the past, but in the past, it was easier to convince yourself that you had control over what was happening around you, even if it was not true.’ (PS, Participant 24)

Almost the same number of participants (*n* = 58, 29.4%) mentioned that their coping strategies had improved and that exercise and family and friends became more important during the COVID-19 pandemic (‘I have more time to exercise so I do it more frequently. I rely much more on friends and classmates for support’ [OTS 11]). About a third of participants (*n* = 58, 30%) revealed that their coping ability had declined. These were the participants involved medical student (MS); nursing student (NS), occupational therapy student (OS); and a physiotherapy student (PS):

‘Before the pandemic I would go for coffee with a friend or visit my family for the weekend, in addition to exercising. Unfortunately, this has not been possible. I would also exercise with friends which I am unable to do at the moment.’ (DS, Participant 2)

Participants revealed that while they did not need medication before the pandemic to deal with stress, they had started using medication to cope.

### Support provided or needed

Participants in this study indicated that additional support ranged from improved communication strategies, empathy, structure, time management skills, and academic and emotional support to cope and function in unfamiliar circumstances. Varied responses were received regarding the support provided by the institution during the COVID-19 pandemic. Some negative responses indicated that more could be done to support students, as illustrated in the next quote:

‘Emotional support every now and then just to be checked up whether everything is still okay. This is a very difficult time for everyone and a little emotional support can go a long way.’ (OS, Participant 158)

On the contrary, some participants were satisfied with the support provided and mentioned:

‘I do not feel like there is anything more that could be done to assist us. Everything we need is being provided already’. (PS, Participant 24)

Other responses linked to online learning and clinical learning, specifically, are included in Table 2.

**TABLE 2:** Support received.

Positive feedback	Negative feedback
**Support from the faculty during the online learning period**	
‘Assistance from the (xxx) department was brilliant. I really thought it was well organized and as interactive as possible which was great as that is what I missed most about being in physical class.’ (PS, Participant 36)	‘During the lockdown the support was somewhat poor. There was a large amount of work that needed to be covered with little guidance from some lecturers, however, this varied greatly depending on the module leader.’ (MS, Participant 138)
‘Extremely well. Everyone is helpful and caring. The university is doing their very best and we really get all the help we need.’ (MS, Participant 102)	‘A weird experience, some lecturers did very well with communication and weekly planning whilst some did not seem to care about communication factor.’ (DS, Participant 193)
**Support received in the clinical setting during the COVID-19 pandemic**	
‘Helpful as we received protective things when we got here. Orientation was given and there is still continuation of supplies when we need them. There has been an enormous support with mental health and being supplied with contacts to reach out.’ (NS, Participant 160)	‘In some facilities it was good. In other not so much.’ (NS, Participant 87)
‘Preceptors were very helpful and considerate of us. They assured us everytime and made us feel at ease and safe.’ (NS, Participant 98)	

Participants mostly responded positively to the support provided during the COVID-19 pandemic at the institution including individual academic support sessions; identifying high-risk students, and referral to social workers, psychologists and psychiatrists.

A specific investigation was done into communication, as a major support strategy, within this study. In response to the open-ended question on the platform of choice for communication from the faculty, participants indicated their choice of mode and frequency of communication. The majority of participants indicated that they preferred online communication from the university, faculty or department, either via Blackboard, email, SMS or WhatsApp. A few participants mentioned a preference for face-to-face communication. Most participants (*n* = 128, 65.3%) indicated that they preferred weekly communication, with smaller numbers indicating a preference for daily (*n* = 29, 14.8%) or bi-weekly (*n* = 26, 13.3%) communication. Only 13 (6.6%) participants indicated preferring monthly communication.

In this study, support in the form of effective communication strategies and the frequency thereof (weekly), and mode of communication (online) came to the fore. Before the COVID-19 pandemic, communication mostly happened face to face, negating students’ other communication needs. In a post-COVID-19 healthcare education setting, integrating various communication strategies might support undergraduate healthcare students academically and psychologically.

## Discussion

This study aimed to investigate undergraduate healthcare students’ psychological well-being, stressors, support needs and coping strategies amid COVID-19. Information on students’ mental health and support needs during challenging times such as the COVID-19 pandemic and as linked to their level of study enables universities to plan support strategies deliberately and effectively (Visser & Law-Van Wyk 2021:241).

There were mixed results on how participants in this study viewed emotional experiences to online learning and the clinical environment. One of the most momentous changes during the hard lockdown of the COVID-19 pandemic was the move to online learning (Gordon et al. 2020). Participants in this study inevitably linked psychological well-being and emotional experiences to the online teaching and learning platform. In line with the findings in this study, multiple authors have reported on the positive and negative experiences with online and blended learning since the COVID-19 pandemic (Connolly & Abdalla 2022:1; Gordon et al. 2020:1; Rafi et al. 2022:1; Saber et al. 2022:1). This has resulted in the publication of, among others, guidelines for developing blended curricula in medical education (Rafi et al. 2022:1), best practice examples of moving healthcare education from traditional face-to-face teaching to blended learning (Tuthill, et al. 2022:1–11), and the dissemination of research on the challenges and barriers of blended learning in healthcare education (Saber, et al. 2022:8). From the university where this study was conducted, limited documentation on online and blended learning has been published, even though several blended learning practices have continued beyond the COVID-19 pandemic. Healthcare educators, at this and other institutions, can build on the positive feedback regarding online and blended learning received from students during the COVID-19 pandemic, as presented in this article, to implement novel and innovative strategies for future undergraduate healthcare teaching and learning practices. The negative experiences can also be utilised to ensure that the necessary support structures are included in online and blended teaching and learning environments, at this and other institutions, to ensure that students’ experiences are more positive. Atwa et al. (2022) in a study among medical students and staff also identified that the value blended learning could hold for healthcare education post-COVID-19, as in this study, but noted that certain aspects of face-to-face healthcare education are ‘irreplaceable’. The participants in this study reported a diverse emotional experience on their clinical training during the time of the study. Clarke et al. (2023:7) reported similar mixed findings on the impact of COVID-19 on the clinical training.

Healthcare students experienced pre-pandemic stress. These included stressors related to a variety of factors such as academic workload. However, the stressors related to the pandemic appeared unexpectedly. Students used different coping mechanisms to manage the stress. The adaptive and maladaptive coping strategies of undergraduate healthcare students in this study are similar to the conclusions of an earlier study among physiotherapy students at the same institution (Janse van Vuuren, Bodenstein & Nel 2018:1). In a study investigating resilience and coping strategies among medical students at the same institution, most students reported academic stress that was associated with lower resilience (Van der Merwe et al. 2020:1). These authors reported that most students had higher resilience scores associated with using adaptive coping strategies, for example, instrumental or emotional support, while fewer students used dysfunctional strategies, for example, substance abuse associated with lower resilience. During the COVID-19 pandemic, and as reported in the current study, the importance of exercise, friends and family was highlighted, indicating the undergraduate students’ inclination to use adaptive coping strategies.

The students who participated in the study mostly reported that their support needs were met. They were satisfied with the communication strategies from the institution that gave information on various support available. This finding is supported by the results from a study conducted among 118 students at a Croatian university, also indicating their satisfaction with communication between students and academics during the COVID-19 pandemic (Juraković, Tatković & Radulović 2022). The Croatian researchers attributed the positiveness of students relating to communication to academics bringing a positive energy into the online environment during the pandemic and assuring that students remained on track to achieving their academic outcomes.

Recommendations for further research emanating from this study include triangulating data from this study with academic performance data among the cohort investigated to ascertain the impact of the pandemic and its associated stressors on academic performance. Healthcare students returned to campus much earlier after the hard lockdown than their counterparts in other fields of study. Therefore, it would be valuable to investigate psychological well-being and coping strategies in the latter student cohorts who spent more time away from campus without the benefit of peer-to-peer interaction as a coping strategy.

## Limitations

This single-site cross-sectional descriptive study is limited by the low response rate by undergraduate healthcare students, despite multiple reminders to encourage participation. The low response rate could be attributed to online survey fatigue as students were inundated with requests for survey participation during the COVID-19 pandemic. Generalisation of study findings is limited because of the possible bias inherent to self-administered questionnaires using a census sampling method.

## Conclusion

Undergraduate healthcare students experienced academic, financial and psychological stress during the COVID-19 pandemic upon their return to campus and clinical training after the hard lockdown period. They reported positive and negative perceptions about the emotional and other support provided and indicated a preference for weekly, online communication. Most students used adaptive coping strategies mirroring existing mechanisms even before the pandemic. In addition, sustainable, creative and sensitive support from higher education institutions is imperative to improve the success and well-being of undergraduate healthcare students to leave no student behind. Higher education institutions should provide students with continued emotional, academic and social support to foster psychological well-being and address individual support needs. Such a collective responsibility requires an open and collaborative learning environment that enables students to learn and thrive. This learning environment could ultimately contribute to academic success and better patient outcomes. Findings emphasise the need for higher education institutions, especially those involved in training healthcare professionals, to provide students with continued support and training to engage in healthy coping behaviours and foster psychological well-being. The findings from this study could inform such future student support initiatives.
